# SURE 2.0 – New release of the worldwide database of surface ruptures for fault displacement hazard analyses

**DOI:** 10.1038/s41597-022-01835-z

**Published:** 2022-11-26

**Authors:** Fiia Nurminen, Stéphane Baize, Paolo Boncio, Anna Maria Blumetti, Francesca R. Cinti, Riccardo Civico, Luca Guerrieri

**Affiliations:** 1 Department DiSPuTer, Università “G. d’Annunzio” Chieti-Pescara, Chieti, Italy; now at RINA Consulting S.p.A, Milan, Italy; 2 IRSN – Institut de Radioprotection et de Sûreté Nucléaire, Fontenay-aux-Roses, France; 3 Department of Engineering and Geology, Università “G. d’Annunzio” Chieti-Pescara, Chieti, Italy; 4 ISPRA – Istituto Superiore per la Protezione e la Ricerca Ambientale, Roma, Italy; 5 INGV – Istituto Nazionale di Geofisica e Vulcanologia, Roma, Italy

**Keywords:** Natural hazards, Solid Earth sciences, Tectonics

## Abstract

Surface rupturing data from the historical earthquakes is used for obtaining empirical regression parameters for fault displacement hazard assessment. This paper represents an additional compilation and analysis effort, extending the first version of the SUrface Ruptures due to Earthquake (SURE) database. This new release contains slip measurements and mapped surface rupture traces of 50 surface rupturing earthquakes of reverse, normal, and strike-slip kinematics occurred all over the world between 1872 and 2019. As a novelty, a ranking scheme of the rupture features is applied to all the traces and slip measurements in the database. Fault ranking introduces geology as a primary analysis tool and allows the end user to obtain regression parameters suitable for the specific geological conditions at the site of interest. SURE 2.0 dataset consists of a table containing the background information about each earthquake, a table containing the slip measurement data of each event, and a joint shapefile containing all the surface rupture traces of the events in the database.

## Abstract (summary)

**Table:** 

Measurement(s)	Surface rupturing due to earthquakes
Technology Type(s)	georeferencing field observation data

## Background & Summary

Earthquake related hazards to human activities and in particular to the infrastructure are not only due to the ground shaking, but also the permanent surface deformation due to surface faulting. Several historical earthquakes have taught how an infrastructure may be strongly damaged by major dislocations of the surface. While it is not possible to predict the earthquake occurrence timewise, the zones that are most likely to undergo surface faulting can be anticipated when the active fault zones are known. In order to predict the distribution and magnitude of surface faulting, empirical parameters are derived from data of historical earthquakes. In particular, probabilistic fault displacement hazard analysis (PFDHA) can be used for addressing the surface rupturing threat^1^, especially in areas where fault zone avoidance cannot be applied because it is not feasible, or because the fault zone is discovered only after building.

The first release of the SUrface Ruptures due to Earthquakes (SURE, hereafter SURE 2020) database was published in 2020^2^. This was the first attempt to gather the surface rupturing data of historical and modern earthquakes into one unified and homogenous dataset. This kind of data is essential for analyses aimed at estimating the fault displacement related hazard in areas and for infrastructure located near and/or on active fault zones. Fault displacement hazard analysis is used for estimating the occurrence of co-seismic fault displacement at the ground surface, and the approach is based on regression parameters describing the spatial distribution of amount and occurrence of surface rupturing. The strength of such a database is in the wealth of good quality data organized homogenously, and more  the events are included, the more accurately the eventual hazard calculations and the corresponding uncertainties can be estimated.

SURE database is also an initiative for establishing a standardized format for reporting the surface-faulting data, as the slip components of historical earthquakes have been communicated in miscellaneous ways. Thus, we have put effort into converting the reported data from various sources into a new, updated SURE 2.0 format. The database with slip components has been designed to be applicable to any geological setting, and one of the purposes of forming such a structure has been its usability in future field working so that the slip components would be measured and reported directly in a format that is compatible  with other such datasets.

This new release of the database, SURE 2.0^3^, is a revised version both by its structure and the events included. Compared to the previous release, several updates and enhancements have been done. Fault mapping accuracy has increased in most cases, and as a major novelty, fault ranking has been applied to all the events. Fault ranking introduces geological knowledge to the database by distinguishing surface ruptures as a function of the causative geological structure, more of which in the following chapter. This enables obtaining regression parameters based on categorization of surface faulting. We have also added several new parameters into the database, and the slip observation points have been linked to the corresponding ruptures in the shapefile with their identification parameter. The overall homogeneity of the initial dataset (SURE 2020) has been re-evaluated and some cases have been left out from this release based on quality selection criteria. In order to ensure regular updating, data can be found on Zenodo, and the scientific community is invited to interact and implement the database by suggesting new data using the form available in the Zenodo repository^3^.

## Methods

### From SURE 2020 to a robust database for FDHA modelling

SURE database is constructed from maps of rupture traces and tables of observation points from various sources. We have combined data of a single event from different sources when available, and brought together data of various format. Maps of different sources were georeferenced as accurately as possible, using also the satellite imagery. For the sake of the coherency of SURE database, the rupture traces were matched to the observation points given with longitudinal and latitudinal coordinates. The events chosen to SURE database cover a rather wide range of magnitude and kinematics. The database structure is designed to host all the parameters controlling the surface rupture pattern and the inputs of the surface geology, but these are largely unfilled in the current release due to lack of original data. The cases in the current release of SURE database (SURE 2.0) are the ones of the previous release^2^ revised, accompanied by the dataset of reverse earthquakes^4^ and some new normal and strike-slip earthquakes not published before in this format.

The cases included in the first release of the SURE database have been re-evaluated in detail for their completeness, accuracy, and consistency. A quality check has been performed to all the events in the database, and even if the resulting databases have some differences, this part of the work was aligned with the work of Fault Displacement Hazard Initiative (FDHI) database^5^. Case completeness was evaluated with respect to good quality data available: several cases, which in SURE 2020 database were compiled from a single bibliographical source were integrated with data from other sources. Especially for the largest events, we tried to include detailed data from the whole length of the principal rupture trace. Accuracy refers both to the spatial accuracy of the measurement or rupture trace location, and the precision of the reported slip measurement. The accuracy of the slip measurement was reviewed also as consistency between multiple data sources, and the variation of the slip measurements and corresponding uncertainties were reported when available.

Basically, the mapping accuracy was improved for several cases by using more detailed georeferenced maps, LiDAR digital elevation models, satellite imagery, and including new input data. In many cases, the geometry of the original data was revised for improving the level of details and the rupture line to data point association. The accuracy of the measurement position was assumed higher than the one of the rupture traces, thus rupture trace accuracy may have been increased by moving the rupture traces or their vertices to points of measurements. Mismatching between points and traces can be caused by several reasons, including coordinates’ accuracy issues between rupture trace shapefiles and point coordinates given in tables (e.g., number of decimals), variety of data sources and the mapping referencing used (for several cases multiple sources by various working groups were utilized), mapping scale used by the first-hand authors, or errors in traces in the original papers. The major changes and updates, including the reasoning behind the decision-making, to the surface faulting data in SURE 2.0 compared to the previous version are detailed in the Database file Notes.txt.

### New and updated content

The SURE 2.0 database contains data from 50 crustal earthquakes (hypocentre depth ≤ 25 km), of which 20 occurred in the US, 5 in Australia, 3 in Greece, Italy and Japan, 2 in Mexico, New Zealand and Peru, and 1 in Algeria, Armenia, China, Ecuador, France, India, Kyrgyzstan, Pakistan, Taiwan and Turkey. Three main types of kinematics are roughly evenly represented: there are 16 strike-slip, 18 normal, and 16 reverse earthquakes in the database. There are 8 events of magnitude M_w_ < 6, the smallest surface rupturing earthquakes in the database being M_w_ 4.9 2019 Le Teil, France, and M_w_ 5.0 2010 Pisayambo, Ecuador and Calingiri, Australia earthquakes. There are 24 medium-large earthquakes of magnitude 6 ≤ M_w_ < 7, and 18 earthquakes of magnitude 7 ≤ M_w_, the largest events being M_w_ 7.9 2002 Denali, USA, and 2008 Wenchuan, China earthquakes. The oldest earthquakes are 1872 Owens Valley, USA, and 1887 Sonora, Mexico, earthquakes. 32 of the events in the database occurred in the previous century, and 18 in the past 20 years. Naturally, the variety in earthquake magnitudes is wider when approaching the present day, as of the historical events only the largest size were studied with an adequate precision even decades after the actual event.

After a careful review throughout the SURE 2020 dataset, the minimum requirements of the updated dataset structure were not met for all the events in the original dataset at the time of releasing SURE 2.0. In general, the minimum requirements include the presence of a principal rupture and some slip measurements, the evidence of distributed ruptures, and the availability of georeferenced datasets with sufficient accuracy in literature. It is important that the two main components of the database, i.e., rupture traces and slip measurements, are present in the published references, because they allow to derive the two associated members of fault-displacement hazard, i. e. probability of principal or distributed surface rupturing, and exceedance of a specific displacement value. With respect to the first release of the database, we also focused on broadening the magnitude range of reported cases (down to below M_w_ 5), and including more of the reverse faulting earthquakes. We also paid attention to share equitably the number of events per type of the focal mechanism. In particular, the 1891 Nobi, 1896 Rikuu, 1918 Omachi, 1927 North Tango, 1930 North Izu, 1938 Kussharo, 1939 Oga, 1943 Tottori, 1944 La Laja, 1945 Mikawa, 1959 Deshibori, 1974 Izu Peninsula Bay, 1978 Izu Oshima offshore, 1984 Nagano Pref. West, 1998 Iwate Pref., 2000 Tottori Pref., 2004 Niigata Pref., and 2008 Iwate-Miyagi earthquakes are events included in the original dataset^2^ but are not yet in SURE 2.0 format. New events have been added in SURE 2.0, such as the 1946 Ancash, 1950 Fort Sage mountains, 1970 Calingiri, 1975 Oroville, 1978 Thessaloniki, 1980 El Asnam, 1981 Pisia, 1981 Corinth, 1983 Coalinga (Nuñez), 1983 Borah Peak, 1986 Marryat Creek, 1988 Tennant Creek, 1988 Spitak, 1993 Killari, 1999 Chi-Chi, 2005 Kashmir, 2008 Wenchuan, 2012 Pukatja, 2016 Petermann, 2016 Amatrice, 2016 Parina, 2019 Ridgecrest I and II, and 2019 Le Teil earthquakes. The 24 new events are dominantly dip slip earthquakes, 8 of which normal, 14 reverse, and two events of Ridgecrest of strike-slip kinematics. The SURE 2.0 database is destined for being evolutive and new cases will be added into the database in near future (such events could be for example 1952 M_w_ 7.5 Kern County, California; 1905 M_w_ 8.0 Bulnay and 1957 M_w_ 8.0, Gobi-Altai, both in Mongolia; 2014 M_w_ 6.9 Yutian, China; 2016 M_w_ 7.8 Kaikoura, New Zealand).

Among new events, all the thrust earthquakes are from the previously published database of reverse faults^4^, some of which with some revision applied. The rest of the new events are normal faulting earthquakes and have been studied and compiled for SURE 2.0. All the strike-slip earthquakes, and some of the dip-slip earthquakes were already included in SURE 2020, but their data were revised for the SURE 2.0 structure and quality level, some even noticeably compared to the previous. The earthquakes included in the database are listed in Table 1, and their more detailed geological, geodetical, and structural parameters are given in the Database file SURE2.0_Earthquakes.xlsx^3^.

**Table 1:** Main parameters of the 50 earthquakes in SURE 2.0 database.

IdE	Name	M_w_	Longitude (°)	Latitude (°)	Depth (km)	Focal mechanism	References for surface rupturing
18720326	Owens Valley	7.45	−118.1	36.65		SS	^14,15^
18870503	Sonora	7.5	−109.25	30.8		N	^16–18^
19110103	Chon-Kemin	7.7	78.53	43.013	20	R	^19^
19151003	Pleasant Valley	6.8	−117.654	40.258	10	N	^20,21^
19461110	Ancash	7.3	−77.535	−8.41	15	N	^22^
19501214	Fort Sage Mts	5.6	−120.066	40.084		N	^21,23^
19541216	Fairview Peak	7.1	−117.981	39.346	10	N	^21,24^
19541217	Dixie Valley	6.6	−117.704	39.207	15	N	^24^
19590818	Hebgen Lake	7.2	−110.891	44.63	10	N	^21,25–27^
19680409	Borrego mnt	6.6	−116.234	33.058	10	SS	^21,28^
19700310	Calingiri	5.0	116.512	−31.092	1	R	^29,30^
19710209	San Fernando	6.7	−118.442	34.254	10	R	^21,31,32^
19750801	Oroville	5.8	−121.546	39.432	5	N	^21,33^
19780620	Thessaloniki	6.2	23.263	40.775	16	N	^34^
19791015	Imperial Valley	6.5	−115.374	32.752	10	SS	^35^
19800525	Mammoth Lake	6.2	−118.908	37.53	10	N	^36^
19801010	El Asnam	7.1	1.374	36.199	10	R	^11,37–39^
19810224	Pisia	6.6	22.482	38.099	10	N	^40,41^
19810304	Corinth	6.2	23.17	38.176	8	N	^40,41^
19830611	Coalinga (Nuñez)	5.4	−120.459	36.244	5	R	^21,42^
19831028	Borah Peak	6.9	−113.796	44.092	10	N	^36,43^
19860330	Marryat Creek	5.7	132.734	−26.31	5	R	^44–46^
19870302	Edgecumbe	6.5	176.80	−37.89	10	N	^47^
19871124	Superstition Hills	6.5	−115.886	33.011	10	SS	^48,49^
19880122	Tennant Creek	6.6	133.883	−19.792	10	R	^50,51^
19881207	Spitak	6.8	44.146	40.897	10	R	^52,53^
19920628	Landers	7.3	−116.557	34.188	10	SS	^54^
19930929	Killari	6.2	76.615	18.07	10	R	^55^
19950116	Kobe	6.9	135.069	34.59	17.6	SS	^56^
19990817	Izmit	7.6	29.979	40.807	15	SS	^57,58^
19990920	Chi-Chi	7.6	120.813	23.834	25	R	^59–72^
19991016	Hector Mine	7.1	−116.557	34.539	20	SS	^21,73^
20021103	Denali	7.9	−147.597	63.512	12.5	SS	^74^
20051008	Kashmir	7.6	73.649	34.451	15	R	^12,75–77^
20080512	Wenchuan	7.9	103.396	30.98	10	R	^78–93^
20090406	L’Aquila	6.3	13.353	42.368	10	N	^94,95^
20100326	Pisayambo	4.95	−78.32	−1.24	1.5	SS	^96^
20100404	El Mayor-Cucapah	7.2	−115.266	32.348	10	SS	^8,97^
20100903	Darfield	7.2	172.17	−43.53	11	SS	^98,99^
20120323	Pukatja	5.4	131.95	−26.16	4	R	^30,100^
20140824	Napa	6.0	−122.312	38.215	11	SS	^21,101^
20141122	Nagano	6.2	137.888	36.641	9	R	^102–106^
20160415	Kumamoto	7.0	130.77	32.84	12.9	SS	^107,108^
20160520	Petermann	6.1	129.863	−25.642	11.2	R	^30,109^
20160824	Amatrice	6.0	13.251	42.704	7.9	N	^110,111^
20161030	Norcia	6.5	13.11	42.832	10	N	^7,13,110^
20161201	Parina	6.2	−70.827	−15.312	12	R	^112^
20190704	Ridgecrest I	6.4	−117.504	35.705	10.5	SS	^113,114^
20190705	Ridgecrest II	7.1	−117.599	35.77	8	SS	^113,114^
20191111	Le Teil	4.9	4.671	44.518	1.5	R	^115^

To date, the database consists of more than 75 000 individual surface rupture traces and close to 19 000 slip observations. The events with most mapped traces are 2002 M_w_ 7.9 Denali (20 999 ruptures), 1999 M_w_ 7.1 Hector Mine (10 887 ruptures), and 2019 M_w_ 7.1 Ridgecrest 2 (10 875 ruptures) earthquakes. The number of individual rupture traces is also a subject to shapefile drawing accuracy, and large number of rupture traces does not necessarily indicate large cumulative length of surface rupturing. 2002 Denali earthquake is in its own class also in total length of all surface rupturing (656 km), and the second largest earthquakes in that respect resulted roughly half of that rupturing: 1992 M_w_ 7.3 Landers (377 km) and 2008 M_w_ 7.9 Wenchuan (345 km).

The level of detail is quite different across the SURE 2.0 events: in fact, for some events only a few or no displacement measurements are available, while for the most recent events surface faulting parameters are measured in great detail. The largest events in the database in terms of measurement points are 2016 M_w_ 6.5 Norcia (7 903 data points), 2010 M_w_ 7.2 El Mayor-Cucapah (1 603 data points), and 2016 M_w_ 6.0 Amatrice (1 561 data points) earthquakes.

### Rupture ranking

**Fig. 1:**
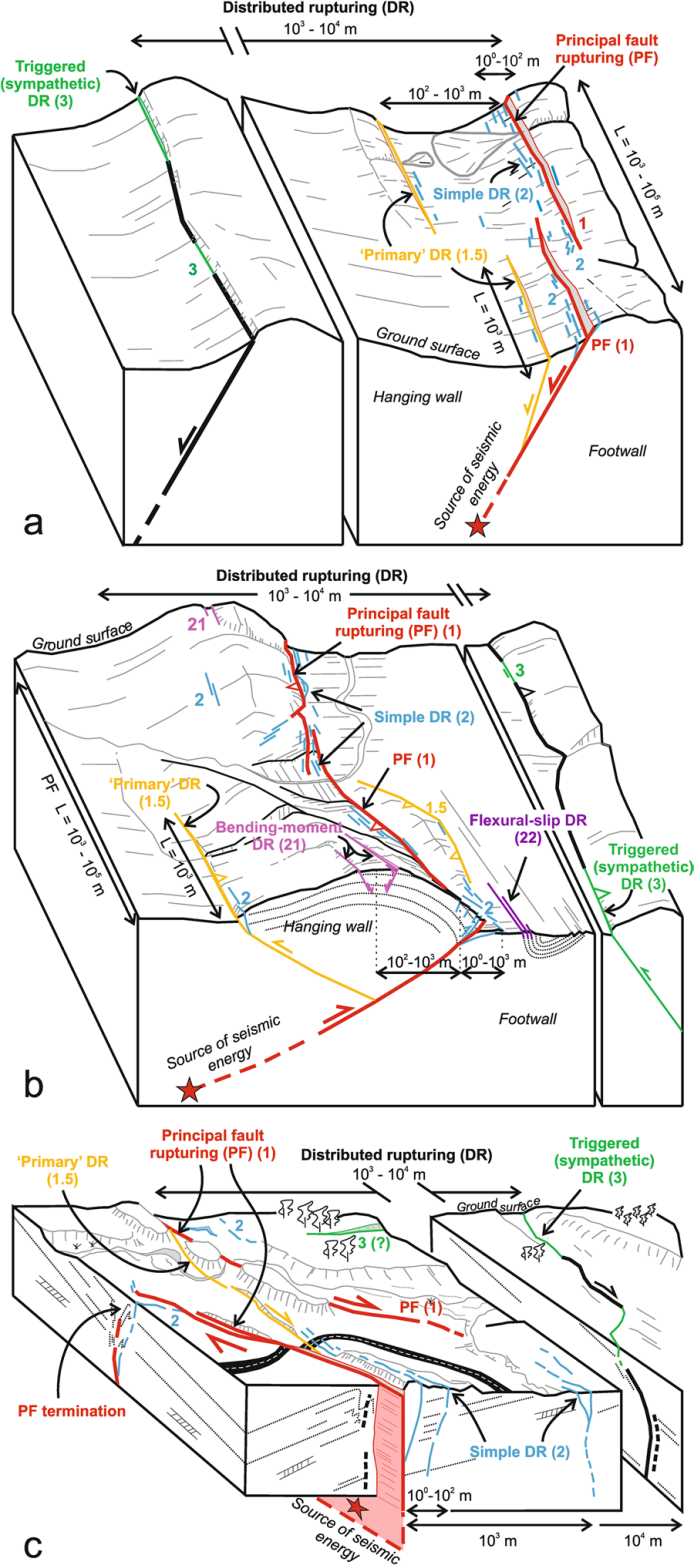
Schematic illustration of fault ranking for (**a**) normal, (**b**) reverse, and (**c**) strike-slip faults. Principal fault (rank 1) is the surface expression of the fault responsible for the earthquake, the other ranking categories refer to various types of distributed rupturing present in different kinematic settings. Primary distributed rupturing (rank 1.5) refers to distributed rupturing along a pre-existing fault that is connected to the principal fault in depth. Simple distributed rupturing (rank 2) is the most common type of distributed rupturing, occurring in unpredictable locations. Triggered rupturing (rank 3) occurs along a pre-existing fault that is not directly connected to the principal fault. Bending-moment (rank 21) and flexural-slip (rank 22) rupturing are both related to large-scale folding associated to reverse faulting.

The most significant improvement to the SURE database in this new release is the surface rupture categorization on the basis of their geological and structural background. When the approach to the probabilistic fault displacement hazard assessment was first introduced, the need for distinguishing the principal fault (hereinafter referred to as PF), i.e., the fault that is responsible for the release of seismic energy during the earthquake, from the distributed rupturing, which refers to all the secondary faulting around the main plane of the slip was emphasized^1^. Further studies^6^ acknowledged the impact of diverse types of distributed rupturing to the empirical regression parameters. These authors distinguished off-fault rupturing by excluding the surface faulting at large distances (>2 km) from the analysis. They stated that such ruptures are of a different nature to the secondary faulting closer to the principal fault. When compiling the SURE 2.0 database, we took this even further, and decided to distinguish the distributed rupturing according to their geological and structural context based on the experience of historical surface rupturing earthquakes. Considering the pre-earthquake studies for example around Monte Vettore in Central Italy, which hosted the 2016 M_w_ 6.5 Norcia earthquake, we can see clearly how some secondary faulting appeared along the pre-existing faults that are subsidiary structures about two kilometres off the Monte Vettore fault^7^. Some off fault rupturing takes place even in remarkable distances, such as the triggered slip that occurred on pre-existing faults in about 100 km distance during the M_w_ 7.2 El Mayor Cucapah earthquake in Mexico^8^. This type of rupturing differs from the most common type of distributed rupturing that appears near the PF with no pre-earthquake background. All the surface rupturing data have been evaluated using a uniform fault ranking scheme (Fig. 1) that includes the categories of distributed ruptures identified in the current database.

According to the ranking scheme, surface rupturing off the principal fault is categorized into rupturing along diverse types of pre-existing structures and to more randomly distributed ruptures in the vicinity of the principal fault. The pre-existing structures can be faults connected to the PF at depth, or separate, unconnected faults, along which displacement is triggered during the earthquake as sympathetic rupturing. These ranking categories can be present in any faulting kinematics. In addition to these ranking categories, large-scale folding related to compressional stress may lead to bending-moment or flexural-slip rupturing in specific conditions around faults of reverse kinematics. Fault ranking is an expert opinion of the Authors, based on those geological criteria. Fault ranking was performed utilizing the earthquake rupture data, but also databases of Quaternary faults, geological maps of the earthquake area, and geological cross-sections.

The ranking scheme applied throughout SURE 2.0 database was first introduced for reverse faults^4^, and similar approach has been applied to normal and strike-slip earthquakes with adequate adaptation. The ranking scheme has been developed first and foremost for fault displacement hazard assessment purposes, so that the hazard at the site of interest can be calculated considering the situation better describing the level of detailed geological knowledge at the site. SURE database user can sort the data accordingly to on-fault (i.e., Principal fault rupturing, rank 1) and off-fault (i.e., secondary, or distributed rupturing, rank other than 1) data, or use the more detailed ranking categories as described below.

#### Ranking categories

We distinguished 6 different faulting types, which are described in detail in the following.

##### Principal fault rupture (PF, *rank 1*)

PF is the surface expression of the movement along the fault plane responsible for the release of the seismic energy during an earthquake. PF has the most continuous surface expression, and it is generally recognized by the highest values of displacement. PF often occurs on fault traces that have the potential to be known and mapped prior to the earthquake, i.e., faults with long-term geologic and geomorphic evidence of activity. The overall strike direction and fault orientation is usually relatively constant throughout the PF strike, even though significant differences from main strike can occur at fault complexities (e.g., bends). SURE database contains only events with principal fault surface expression, thus no blind earthquakes are included.

##### Simple distributed ruptures (*rank 2*)

All the surface rupturing off the principal fault is called distributed rupturing (DR), some of which may take place in kilometres away from the PF trace. In the most common case, it can be categorized as simple distributed rupturing (*rank 2*), by which we mean discontinuous surface breaks around the PF that occurred as a direct response to the movement along the earthquake fault. Simple distributed rupturing occurs rather randomly around the PF trace, and the measured displacements are remarkably lower than of the PF rupturing nearby. The occurrence of simple distributed rupturing is largely guided by the subsurface material, but the surface cover may help or hamper to recognize the distributed rupturing, especially when of small displacement in unconsolidated material. Distributed rupturing is frequent around PF complexities, such as steps and overlapping zones, and in those cases they are often simple DRs. This is the most common DR type in earthquakes of all kinematics.

##### Primary distributed ruptures (*rank 1.5*)

As the fault systems are often complex and interconnected, movement along one fault plane may cause slipping also along some other, pre-existing surrounding structures. In many cases, these types of structures can be recognized and mapped before an earthquake, as there can be geologic and geomorphic evidence of long-term activity on them. In cases when the geological data (e.g., geologic maps and cross-sections) suggest these structures are directly connected to the principal fault at depth, we categorize DR along these pre-existing structures as primary distributed rupturing (*rank 1.5*). As the rupturing occurs along a pre-existing fault plane, these types of DRs are usually more continuous of their surface expression than the *rank 2* simple DR. Also, the amount of slip may be significantly larger than the one of the simple DRs at similar distance to the PF as the slip is facilitated by an existing fault plane, which could be pre-stressed and ready to slip. Primary distributed ruptures may or may not be able to provoke an earthquake by themselves, but they may provoke simple DRs of their own as they rupture with the connected principal fault. Primary distributed rupturing can be identified in earthquakes of all kinematics.

##### Triggered distributed ruptures (*rank 3*)

Rupturing along a pre-existing structure that is not likely to be connected to the PF at depth are categorized as triggered distributed rupturing (*rank 3*). In general, triggered distributed ruptures are the most distant ones from the PF. Typically, triggering causes often a non-linear surface expression of low continuity, i.e., plenty of discontinuous DR traces around a pre-existing fault structure, at distances where no aleatory (*rank 2*) rupturing is present in same density. Triggered DRs are hosted by active faults, but the level of their own activity may vary. In the database we also have cases where *rank 3* rupturing was triggered along faults that acted as principal fault during different events included in the database. Among these, the 2010 M_w_ 7.2 El Mayor-Cucapah that caused triggered rupturing along the fault that had been principal faults of 1987 Superstition Hills, 1979 Imperial Valley, and 1968 Borrego Mountain earthquakes. Triggered DR can be present in any fault kinematics.

##### Bending-moment (B-M, *rank* 21) and flexural-slip (F-S, *rank* 22) ruptures

Compressional stress of reverse faulting may result in large-scale folding of hundreds of meters to kilometres in wavelength, which, in favourable conditions, may lead to bending-moment or flexural-slip rupturing^9,10^. Bending-moment rupturing (*rank 21*) are normal faults that are formed close to the hinge zone of large-scale anticlines on the hanging wall of a reverse fault. Flexural-slip rupturing (*rank 22*) is formed due to differential slip along bedding planes on the limbs of a bedrock fold. In case of large-scale folding, distributed surface rupturing does not occur on pre-existing fault planes that could be traced before an earthquake, but surface ruptures are still less randomly located as the simple DR (*rank 2*) and occur only when fold structures are present.

#### Processing the surface rupturing data

**Fig. 2:**
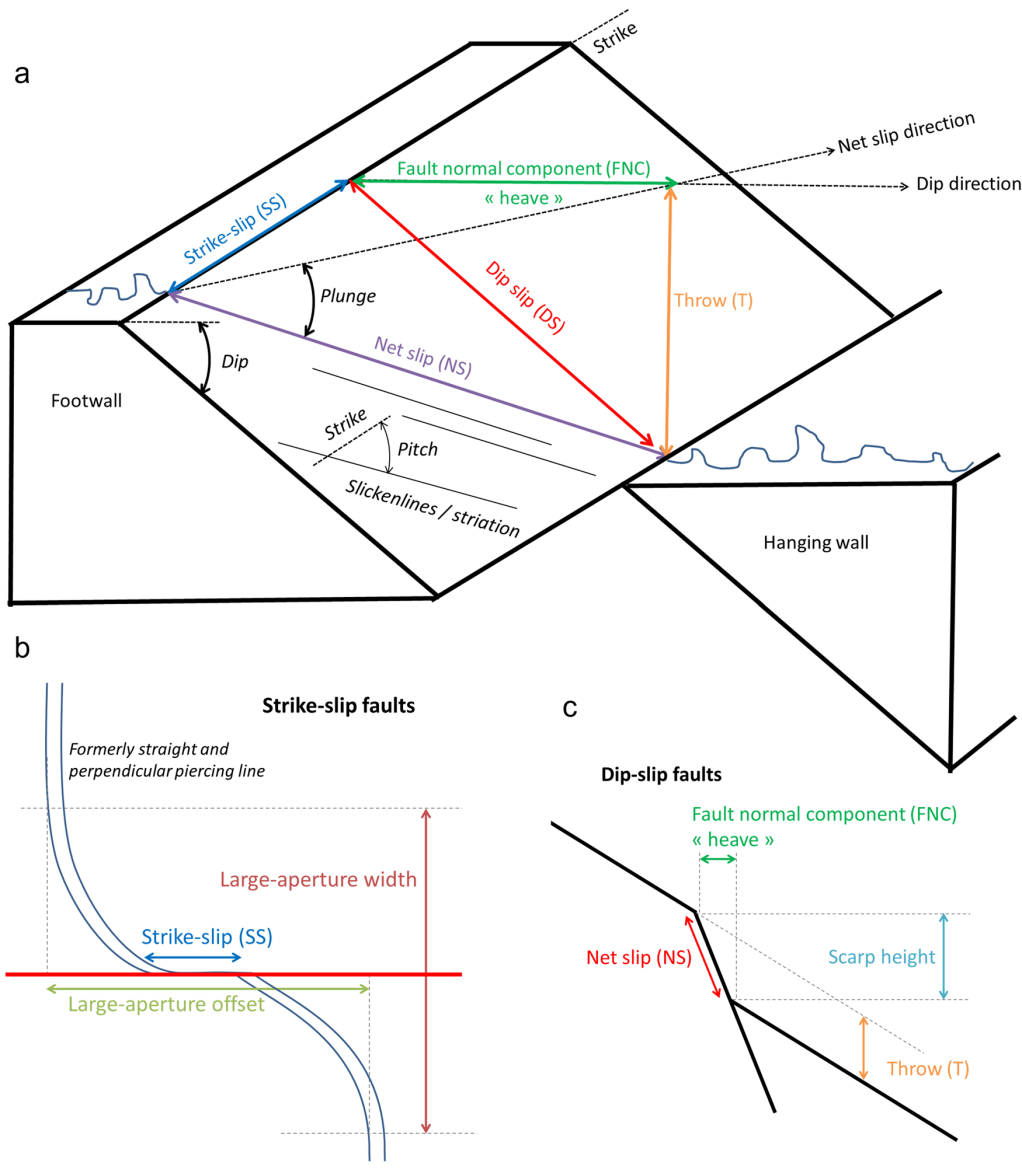
Slip components and the nomenclature used in SURE 2.0 database. (**a**) The slip components and angle and direction parameters shown in perspective view. (**b**) Map view illustrating the parameters related to strike-slip faulting. (**c**) Profile view schematization illustrating the parameters related to dip-slip faulting.

Surface rupturing data from various sources reported according to various standards was collected to the joint database by finding the best corresponding attribute to the data parameters. The nomenclature and slip parameters used in SURE 2.0 database are shown in Fig. 2. In Fig. 2a,the slip parameters are shown for normal fault, but the same parameters are applied to ruptures of all kinematics, as applicable. It should be noticed, however, that the parameters are reported as absolute values in the database, and the direction is indicated by additional parameters. Figure 2b,c illustrate the nomenclature that is specific for strike-slip and dip-slip movement, respectively.

**Fig. 3:**
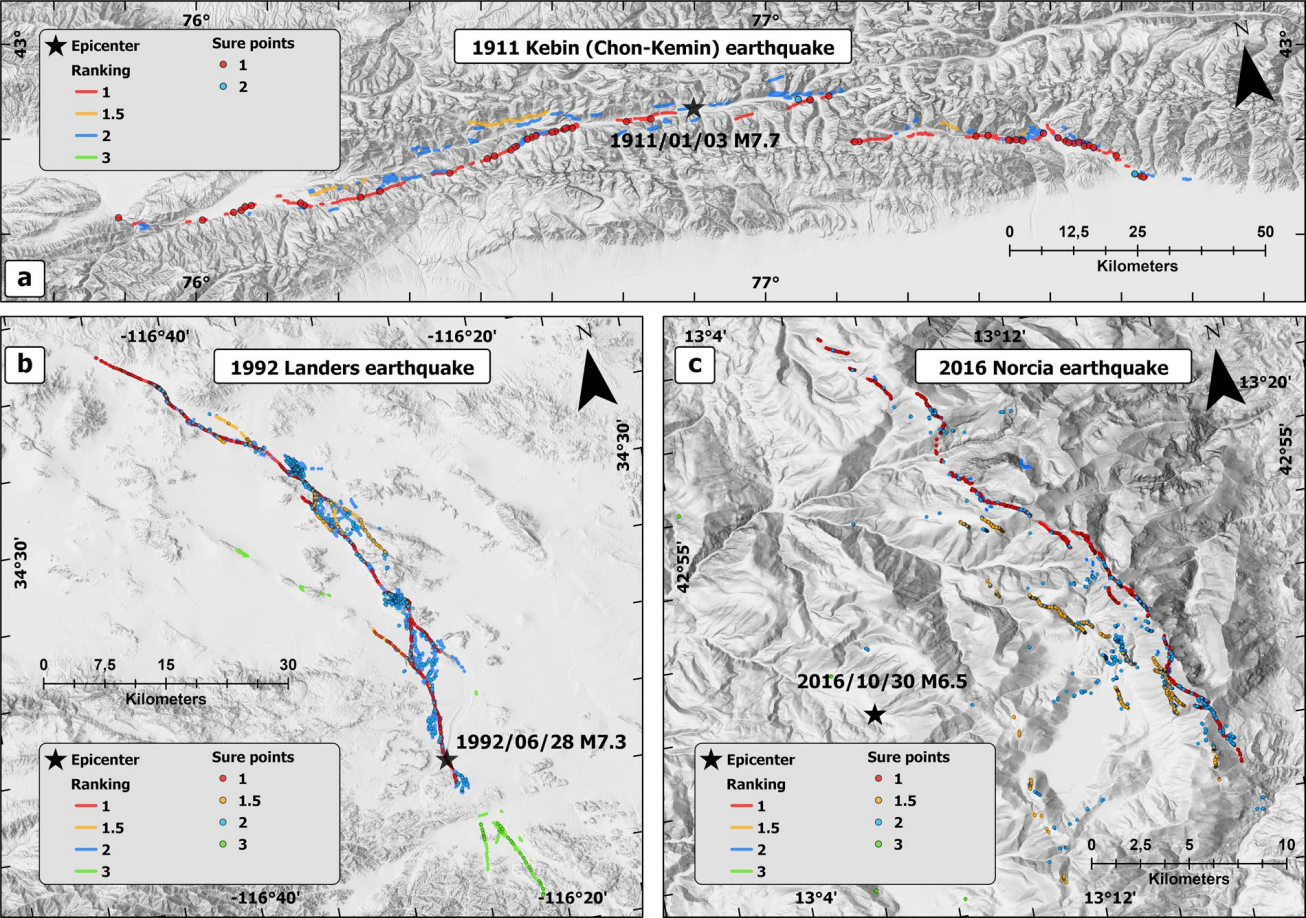
An example of mapped surface ruptures with fault ranking. (**a**) 1911 M_w_ 7.7 Chon-Kemin earthquake of reverse kinematics. (**b**) 1992 M_w_ 7.3 Landers earthquake of strike-slip kinematics. (**c**) 2016 M_w_ 6.5 Norcia earthquake of normal kinematics.

Geological maps and data of fault systems, as well as satellite imagery, were used when available to map the rupture traces as accurately as possible. Some cases were studied by using the published shapefiles, and some surface ruptures were drawn by using georeferenced maps. It is worth noticing that there are some differences in the ways in which the shapefiles are drawn, and the shape continuity vs. discontinuity may vary between the cases. Most of the cases with published rupture shapefiles were considered as such, and the shapefiles drawn from georeferenced maps were obtained working in 1:1000 to 1:500 scale. When talking about an empirical dataset of surface rupturing data from the past 135 years, the continuity of surface rupture traces results affected by: 1) Level of detail in field working surveys. There are cases in which the surface rupturing maps are rather indicative, and other cases in which the level of detail is extreme which has allowed drawing the fault traces in detached segments within a minimal distance from another, just as it appears at the ground surface. Generally, but not always, these latter are the most modern earthquakes. 2) Landscape features and characteristics. The presence of urbanized area or a waterbody can obscure the continuity of the fault structures. The surface rupture traces are brought to SURE database as they were reported by the original sources, but some revisions may have been done also to the shape continuities. An example of surface ruptures of an earthquake of each kinematics is given in Fig. 3. Fault ranking is with similar colours as in Fig. 1.

The objective of applying the detailed ranking scheme to the SURE database is to add supplemental geological information through expert judgement into the compilation. Besides the PF, there is some rank 2 simple distributed rupturing present in every earthquake. Ranking a rupture into any complex type of distributed rupturing categories (*rank 1.5, 21, 22, 3*) is done based on some additional insights from geological and seismological data. Ranking of each case was reviewed by the Authors, and it is based on the most detailed information available at the time of ranking.

In practice, first thing to distinguish from the mapped surface rupturing data was the PF trace, or traces. The objective was to find the most continuous surface expression occupying the major co-seismic displacement. PF surface rupture traces may contain several types of structural complexities and discontinuities, but the general fault orientation, continuity of rupture and amount of displacement were used to guide the decision to define the traces to *rank 1*. From all the often numerous, parallel rupture traces the target was to define one PF trace based on the continuity and the largest slip if possible, other traces being of other ranking categories. Some large reverse and strike-slip earthquakes triggered significant and continuous slip on parallel or branching strands. We assigned both those ruptures as principal fault, meaning that for those cases we have two parallel *rank 1* PF segments.

In some cases, some distributed rupturing stands out by a remarkable displacement or notable continuity in respect to other distributed rupturing in similar distance from the PF. Especially when these surface expressions overlap with some pre-mapped faults, they are ranked as primary distributed rupturing (*rank 1.5*) or triggered distributed rupturing (*rank 3*). If a detailed geological mapping was not done in the area prior to the earthquake in question, fault orientation parameters or geological cross-sections drawn at the site after the earthquake may also have led to these rankings. Triggered DR (*rank 3*) is often distinguished by their remarkable distance to the PF, and visible separation to the other distributed rupturing on the map view. All the discontinuous rupturing observed near or at the triggered ruptures are classified as *rank 3*, as their presence is controlled by the triggering of the pre-existing rupture.

Large-scale folding related DR types are due to specific conditions around a reverse fault. Ranking of both bending-moment (rank 21) and flexural-slip (rank 22) rupturing is based on additional data provided by the first-hand authors. For example, detailed illustrations of the rupturing in various parts of the 1980 M_w_ 7.1 El Asnam earthquake show both bending-moment and flexural-slip distributed rupturing^11^. Similarly, cross-sections of Sar Pain area of 2005 M_w_ 7.6 Kashmir earthquake show the normal rupturing on top of the anticline due to large scale folding^12^.

For all dip-slip earthquakes, all the DRs are positioned either on hanging wall (HW), or on footwall (FW) of the corresponding principal fault. In cases of structural complexities of the principal fault, such as step-overs or other discontinuities, some general rules were applied for systematic processing of the data. DRs occurring in the overlapping zone of dip-slip PF segments are generally assigned in the HW side, except for those DRs very close to the PF trace that they are on the FW side. In cases in which hanging wall vs. footwall is not defined by the nearest PF trace but some other, the identification number of the PF trace, to which the DR is associated with, is defined in its attributes with the identification of the corresponding PF (IdS_PF). At the fault ends, short and discontinuous distributed ruptures are often mapped in fan-like shape away from the PF tip. For these DR traces the hanging wall vs. footwall is defined based on the hypothetical extension of the PF trace from its tip maintaining its direction. Considering that the primary distributed ruptures (*rank 1.5*) can cause simple distributed rupturing of their own, for rank 2 simple DRs the hanging wall vs. footwall parameter was distinguished for two situations: 1. HW vs. FW with respect to the* rank 1* PF (HW_FW_PF), and 2. HW vs. FW with respect to the nearest *rank 1* or *rank 1.5* fault (HW_FW_near). The latter rule is applied within 1 km distance from *rank 1.5* faults; all the DRs further away are associated only with the PF. For example, in dataset of 2016 M_w_ 6.5 Norcia earthquake there are some simple DRs situated on the footwall (HW_FW_near: *FW*) of a *rank 1.5* primary distributed rupture, which itself, its simple DRs included, is on hanging wall of the PF (HW_FW_PF: *HW*). As all the other types of DRs (ranking other than 2) are associated only with the PF, they only have the parameter HW_FW_PF.

Considering that slip can be measured only where some rupturing has been observed, some shapefiles have been modified from initial references for matching the measurement points to ruptures, often by slightly dispositioning the rupture shape (or its vertices), or if no traces are given in the vicinity, by drawing a short fault trace at the point. Some rupture segments may have been repositioned or altered slightly by their vertices according to the coordinates of slip measurement data. Even in these cases the idea is that the overall shape and length follow the one from the original source.

## Data Records

SURE 2.0 database^3^ available in Zenodo repository consists of three parts containing i) the basic parameters of each earthquake in the database (SURE2.0_Earthquakes.xlsx), ii) the displacement data of each event (*IdE_EventName*_SURE2.0_Slip_Obs.xlsx), and iii) the line shapefile providing the geometry of surface ruptures of each earthquake (*IdE_EventName*_SURE2.0_ruptures.shp). Surface displacement data are reported in the database by the slip components in each direction, which are synthetised in Fig. 2. Details of the data structure including the data attributes have been updated with respect to the SURE 2020.

### Basic parameters of the earthquakes

General information of the earthquake: hypocentre related parameters (coordinates, depth, focal mechanism), size (magnitude), time of occurrence, and structural background of each event (Table 2) is gathered into SURE2.0_Earthquakes spreadsheet. The tabular file lists all the events in the database and may represent as a tool for database users to select the events by different parameters.

**Table 2:** Earthquake information.

ID	IdE	Concatenation of earthquake date numbers yyyy/mm/dd in GMT
	Name(hyperlink to USGS)	Usual name of the earthquake
	Region	Country or Flinn–Engdahl regionalization
	Fault system name	From bibliography, if any
Earthquake parameters	Year	Year of the earthquake, in GMT
	Month	Month of the earthquake, in GMT
	Day	Day of earthquake, in GMT
	Longitude	Longitude of the epicentre in decimal degrees
	Latitude	Latitude of the epicentre in decimal degrees
	M_w_	From ISC catalogue or alternative source (recent events)
	Comment on magnitude	
	Depth	Hypocentre depth in km, from ISC catalogue or alternative source (for most recent events)
	Focal mechanism	Dominating kinematics: reverse, normal or strike-slip
	Reference for seismological parameters	
Geological parameters	SRL from geology [km]	Surface rupture length in km, from literature
	MD [m]	Maximum displacement in m, from literature
	AD [m]	Average displacement in m, from literature
	Reference for geological parameters	
Geodesy	Geodesy information	GNSS, InSAR, or other geodesy data provide insight on coseismic surface deformation
	Reference for geodesy	
	Reference for rupture at depth	
General background and fault history	Moho depth [km]	
	Reference for Moho depth	
	Structural background	Structural background of the seismicity (e.g., Basin and Range, Fold-and-Thrust range, Intraplate)
	Inversion tectonics	yes/no
	Köppen classification climate (Rubel and Kottek, 2010)	Basic context in terms of climatic terms (Köppen–Geiger classification)
Fault history	Paleoearthquake history	Paleoseismological record
	Slip rate	From literature, if any
Original data	Reference for surface rupturing	
	Description of implementation process and map scale	

This table summarizes the contents and structure of the Database file SURE2.0 Earthquakes.xlsx^3^. The structure is modified from SURE 2020^2^.

### Ruptures shapefile

The rupture shapefiles contain all the mapped traces of all the events in the database. The rupture traces attribute data contain, as a minimum, earthquake identification number (IdE), iterative number of the rupture (*IdS*), a joint parameter combining these two as an identification of a singular rupture trace (*IdE_IdS*), rupture length, working group ranking (*Comp_rank*), citation of used reference, and a note if the rupture geometry has been revised with respect to the original source (*Geom_rev*). When the rupture is a DR of a dip-slip (normal or reverse) earthquake, a parameter indicating its location either on hanging wall or footwall side of the corresponding primary fault is a compulsory feature in the database. The shapefile attributes and data format used are listed in Table 3.

**Table 3:** Rupture shapefile attribute information.

FIELD ALIAS	CONTENT	DATA FORMAT
**IdE**	Earthquake identification number	*YYYYMMDD*
**IdS**	Iterative number of rupture	A sequential number starting from 1 for each event
**IdE_IdS**	Identification number of the rupture in SURE database	*IdE_IdS*
F_section	Fault section name	free description
**Length**	Rupture length	in meters
Strike	Strike measured in the field	in degrees
Dip	Dip measured in the field	in degrees
Cover_bed	‘b/b’ = rupture in bedrock	*b/b*
	‘b/s’ = rupture between bedrock and sediments	*b/s*
	‘s/s’ = rupture between sediments	*s/s*
F_complex	Fault pattern complexity	free description
Auth_rank	Rupture ranking according to the first-hand authors (see Fig. 1)	*1*
		*2*
		*1.5*
		*21*
		*22*
		3
**Comp_rank**	Rupture ranking by SURE working group (see Fig. 1)	1
		2
		*1.5*
		21
		22
		3
HW_FW_PF *	Location of observed distributed slip respect to the upthrown/downthrown sides of principal fault plane	*HW*
		*FW*
**HW_FW_near**	Location of observed distributed slip respect to the upthrown/downthrown sides of nearest principal fault (rank 1) or primary distributed rupture (rank 1.5) whichever the closest**	*HW*
		*FW*
Paleoeqs	Brief description of paleoseismological record	free description
Slip_rate	Slip rate	
**References**	Citation of used reference	*Author(s), YYYY*
**Geom_Rev**	Rupture geometry being revised with respect to the original source	*yes*
		*no*
IdS_PF*	IdS of the primary fault a distributed rupture is associated to if not the nearest one	

This table summarizes the contents of the attributes associated to the Database files *IdE_EventName*_SURE2.0_ruptures.shp^3^. The structure is modified from SURE 2020^2^.

Mandatory attributes marked in bold.

*Only for distributed rupturing, when applicable.

**Maximum distance in which a primary distributed rupture (*rank 1.5*) is considered the nearest is 1 km.

### Slip observation data

Slip observation data of all the earthquakes in the database is listed in a uniform way in *IdE_Name*_SURE 2.0_Slip_Obs spreadsheets, given separately for each earthquake in the database. The attributes given to the observation points are grouped in 11 subgroups describing the basic information of the observation point, various slip components, fault and site related information, and fault ranking. Each subgroup is better detailed the second row of the tabular file. In addition to this, on the third row of the file we provide the field aliases in a way that is better compatible with the most used programs for geospatial data analysis. The structure and contents of the slip parameter attributes are detailed in Supplementary Table 1. There are some minor updates to the tabular file structure with respect to the SURE 2020 release.

The SURE 2.0 structure enables reporting of all surface rupturing, associated fault and the site related data, and all the slip components with corresponding errors, and minimum and maximum measurements reported at the location. Naturally, the data available varies from point to point, but the minimum information, provided for each point are the identification parameters of i) the earthquake (*IdE*); ii) the rupture segment the slip measurement is related to (*IdS*; from the surface ruptures shapefile attributes), and iii) the measurement point itself (*IdO*). Other mandatory information concerns used references, observation point coordinates (reported in WGS84), compiler ranking, and the information about the possible revision of the geometry. Only the observation points with some reported slip component are included, but no mandatory fields are defined as the slip can be of any direction. Similarly as for the ruptures, if the point is on a distributed rupture of a dip-slip fault, also the positioning either on hanging wall or on footwall is a mandatory parameter.

## Technical Validation

The validation of the database, for each component (traces, slip observation points, earthquake data), was completed through various checking. The earthquake population was initially shared into fault mechanism groups (normal, reverse, strike-slip) and treated and implemented by sub-groups of co-authors, after which the cross-checking sessions were convened. With this, we ensured the accuracy of rupture traces (in respect to the original references) and the matching between one measurement point and a rupture trace. For instance, we corrected erroneous slip values (e.g. correcting “0” values to “no value”), manually re-digitized traces where surprisingly uneven or extremely “segmented” (at a meter scale), and above all verified the consistency of ranking strategy based on geological information all through the database. In a text document available in the Zenodo repository (Notes.txt) we have gathered the notes regarding the data gathering and revision process applied to surface faulting data of each of the earthquake in SURE 2.0 database. In that document we have also listed the updates and modifications compared to the previous version of the database.

### Basic statistics of the database

The SURE 2.0 database consists of 18 885 data points and 75 695 rupture traces totalling 4 481 km of their cumulative length. Data points per event vary massively between the events, the average being 118 points per event. The 2016 M_w_ 6.5 Norcia has the greatest number of data points (7 903), followed by 2010 M_w_ 7.2 El Mayor-Cucapah with 1 603 points, the 2016 M_w_ 6.0 Amatrice with 1 561 data points, and 1992 M_w_ 7.3 Landers earthquake with 1 123 data points. All the rest of the events have less than 550 points per event, and the least number of data points are reported from 1988 M_w_ 6.6 Tennant Creek (6 data points), 1950 M_w_ 5.6 Fort Sage Mountain (3 data points), and 1946 M_w_ 7.3 Ancash earthquakes (0 data points). Differences in number of slip observation points seem to depend not only on the size of the earthquake, but also the number of the scientists involved in the field: in Central Italy earthquakes of 2016 there were numerous research groups collecting the slip observation data which were gathered into joint Open EMERGEO database^7,13^. We can speculate also that it is easier to measure the slip for dip-slip offsets than strike-slip ones, which may partially explain the opposite balance between the slip observation and number of traces for Ridgecrest when compared to Norcia earthquake, even if they both are recent events studied in great detail in the field.

**Fig. 4:**
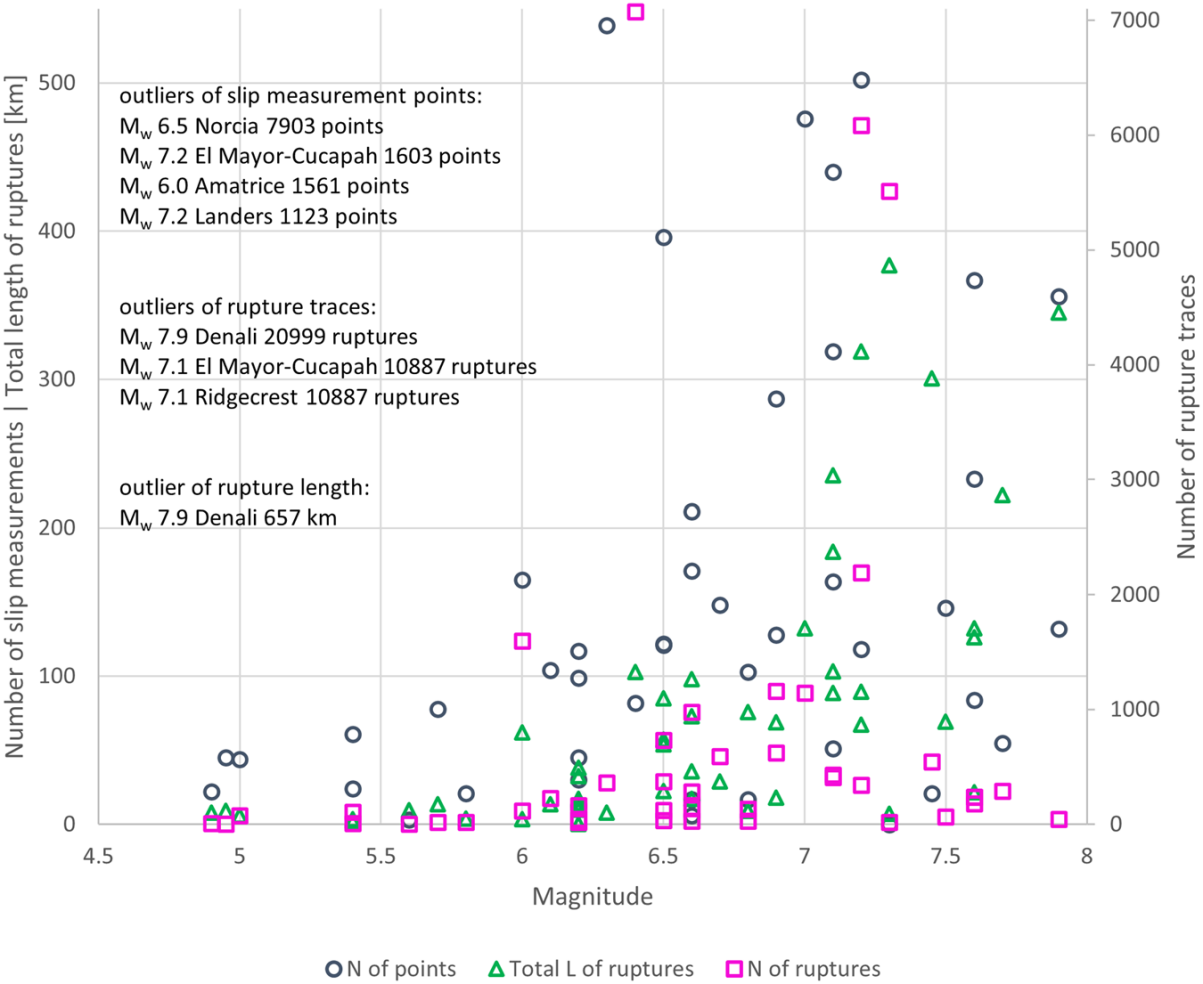
Number of slip measurement points and rupture traces plotted against the magnitude of the earthquake. Both y-axes are cut, leaving some remarkably high values out from this plot: for points M_w_ 6.5 Norcia with 7 903 points, M_w_ 7.2 El Mayor-Cucapah with 1 603 points, M_w_ 6.0 Amatrice with 1 561 points, and M_w_ 7.2 Landers with 1 123 points; for rupture traces M_w_ 7.9 Denali with 20 999 traces, M_w_ 7.1 Hector Mine with 10 887 traces, and M_w_ 7.1 Ridgecrest 2 earthquake with 10 875 traces; and M_w_ 7.9 Denali earthquake with the total length of the surface ruptures of 657 kilometres.

Similarly as with the number of slip measurements, there is a rising trend in number of slip measurements with the increasing magnitude of the earthquake (Fig. 4). The largest number of individual rupture traces in the database are from 2002 M_w_ 7.9 Denali (20 999 rupture traces), 1999 M_w_ 7.1 Hector Mine (10 887 ruptures), and 2019 M_w_ 7.1 and M_w_ 6.4 Ridgecrest earthquakes (10 875 and 7 074 ruptures, respectively), all the rest of the events having less than 7 000 rupture traces per event. For the sake of readability of the plot in Fig. 4, both the vertical axes were cut leaving the highest values of all the plotted categories out. The most numerous data is from the most recent events in the database.

**Fig. 5:**
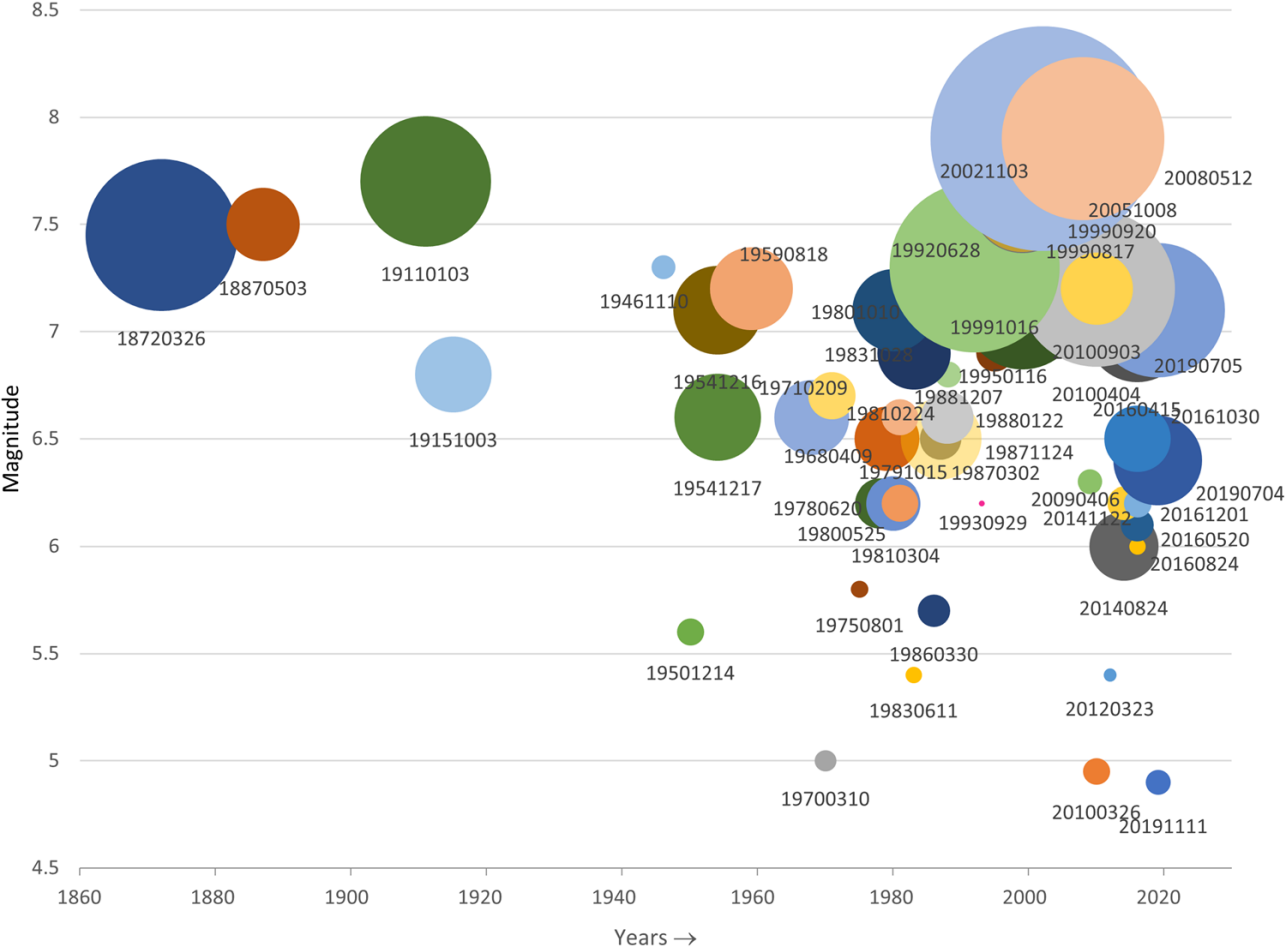
The events in the SURE 2.0 database according to their date of occurrence (x-axis) and magnitude of the earthquake (y-axis). Size of circle is proportional to the total length of the surface rupture traces in the database (all ranking categories included). Notice, that in 1987 there were two earthquakes of M_w_ 6.5: Edgecumbe with cumulative ruptures length of 22.5 km, and Superstition Hills with cumulative ruptures length of 85.1 kilometres, which are plotted here one on top of another. Also, 1999 earthquakes of Izmit and Chi-Chi are both of M_w_ 7.6, with cumulative ruptures lengths of 132.2 and 126.1 kilometres, respectively.

We introduce the total length of the surface ruptures as a parameter to represent the extension of surface faulting and to compare the events in the database. By total length of the surface ruptures we mean here the sum of the lengths of the rupture traces of any ranking category. This value should not be confused with the commonly used parameter of surface rupture length (SRL), which refers to the end-to-end length of the principal fault of the event, usually measured without considering the fault trace complexities. Total length of the ruptures varies largely from event to event, but naturally greater total length corresponds with the higher magnitude of the event (Fig. 5). In addition, we notice that for the events of same magnitude, total rupture length increases with the year of occurrence, as a clear signal of greater precision in the co-seismic surveys. For comparing the datasets of the events in the database, we prefer the total length over the number of traces, as the latter depends largely on the continuity of the trace mapping and the level of detail at which the shapefile of the rupture was constructed.

### Fault ranking reveals the differences within distributed rupturing

**Fig. 6:**
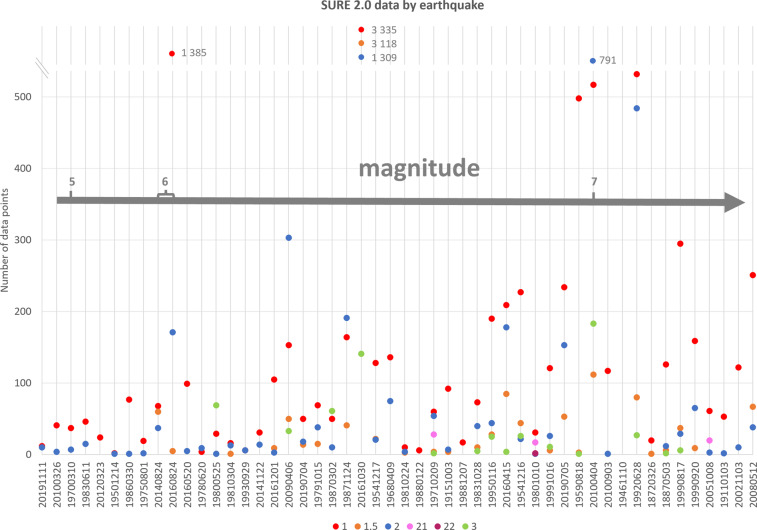
The number of the data points in SURE 2.0 database according to their ranking. The vertical axis is cut at 540. The markers above the cut are accompanied with a label of the number of the points of the ranking category in question; the markers above the cut are not plotted in scale. The earthquakes are organised according to their magnitude, magnitude increasing to the right on horizontal axis.

**Fig. 7:**
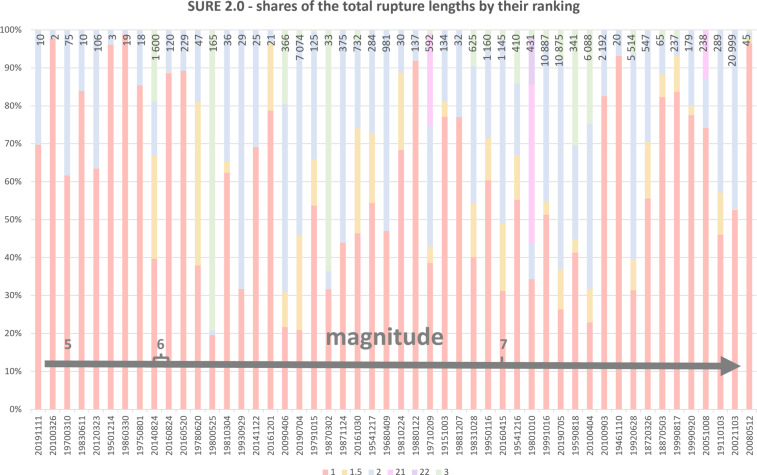
Shares of the total rupture lengths by ranking categories. The number above the stacks is the total number of rupture traces in the database (all ranking categories included). The events are organized to increasing magnitude from left to right. Principal fault rupturing (*rank 1*) represents about 60% of all the rupturing per event in average, and for most of the cases, simple distributed rupturing (*rank 2*) represents 10–40% of total. Complex DR types (*ranking 1.5, 3, 21* or *22*) are identified in 35 cases out of 50.

Analysis of distributed rupturing according to their ranking reveals differences between the rupturing types (Figs. 6 and 7). On a case-by-case basis, in average *rank 1* PF ruptures represent roughly 70% of the measurement point data, and about 60% of the total surface ruptures length. Most of the cases set between 50 and 90%, roughly, in measurement points, and between 40 and 80% in total rupture length, but there are some cases in which PF represents only one fifth of the total, or close to 100% of the data. As the PF is defined as the most continuous surface expression, it is not surprising that the share of the PF length is in average only a bit over a third of the total number of ruptures by the case.

*Rank 2* simple DR is the most common type of distributed rupturing in the database. However, not in all cases these ruptures have related slip measurement data. In average, *rank 2* DRs represent almost one fourth of the total length of the ruptures, and about 15% of the measurement points. Thus, this ranking category represents rupturing of high number of occurrence but large discontinuity, and small, often even immeasurable displacements (especially in the strike-slip environment). There are 15 cases in total, in which beside the *rank 1*, only *rank 2* type of DR was recognized. Most of these cases represent the smallest class of magnitude, but there are also some larger earthquakes where no complex types of DRs were identified, probably also due to lacks in field observations. For example, 1946 M_w_ 7.3 Ancash earthquake is very likely to be under-sampled especially for its distributed rupturing. The 1988 M_w_ 6.8 Spitak earthquake present also relatively little surface rupturing data considering the size of the earthquake.

Primary distributed rupturing (*rank 1.5*) has been observed in 30 earthquakes, of which the smallest of their magnitude are 2014 M_w_ 6.0 Napa and 2016 M_w_ 6.0 Amatrice earthquakes. By definition, appearance of this kind of surface rupturing depends on the existing fault structures and its recognition depends mainly on the availability of detailed geological data and accurate topographic maps.

Bending-moment rupturing (rank 21) is a very local phenomenon that was detected only in three events of reverse kinematics in the current database: 1971 M_w_ 6.7 San Fernando, 1980 M_w_ 7.1 El Asnam, and 2005 M_w_ 7.6 Kashmir earthquakes. Large-scale co-seismic folding causes numerous breaks on the anticline, thus in events where bending-moment distributed rupturing is observed, the number of these ruptures represent a significant share of all the distributed rupturing traces of that event (from 30 to 71%), and a remarkable part of the total length of the ruptures by their ranking (from 40 to 64%). These areas are also very well studied in terms of slip measurements, and there are numerous* rank 21* measurement points in all these cases. Of the events in the current database, flexural-slip distributed rupturing (rank 22) was observed only in the 1980 El Asnam earthquake. It was seen important to separate these from the simple distributed data as the occurrence of this kind of rupturing is so heavily guided by the bedding plane slip.

## Usage Notes

Putting together data from various sources has not always been easy due to various reporting standards used in the field observations. For the sake of reliability, only the data we could convert to SURE standard with a minimal uncertainty were included, and in some cases some additional information might have been left out. Since the first-hand surveyors better know what type of measurements were done, we invite the scientific community to add and update the data in the database. Also, data from new events is warmly welcome. New and updated cases can be suggested in the repository, and after a revision by the Authors they will be integrated to the merged database in its next update.

### Adding new data

For contributing to the database by adding data of new events we invite the contributors to familiarize themselves with the slip observations database structure (Supplementary Table 1), and to notice the minimum requisites that should be filled in. This includes familiarizing with the ranking scheme, in order to provide the compiler ranking. Slip observation data is filled in an empty file (SURE2.0_Slip_Obs_empty.xlsx) that can be found from Zenodo repository, and is to be saved as “*IdE_EventName*_Slip_Obs.xls”, *IdE* indicating the identification number of the earthquake, formed from year, month and day of the event. The corresponding shapefile should be constructed with the SURE 2.0 attributes (Table 3) and saved as Polyline in WGS84, named as “*IdE_EventName*_SURE2.0_ruptures.shp”. Basic information of the event is filled in an empty template of SURE2.0_Earthquakes_empty.xlsx, and saved as “*IdE_EventName*_Earthquakes.xls”. Used references are cited in tables and attributes as indicated, and the full citations are saved in a simple text file saved as “*IdE_EventName*_references.txt”.

### Reviewing existing data

In case of updating the data already in the merged database, we invite the contributors to copy the data of the earthquake to be updated, to make the updates and prepare separate flat file for slip observations and corresponding shapefile following the steps described for new cases. The files are saved to the folder of the cases to be reviewed, and update to the existing cases will be done as new cases are integrated to the merged database. In case of reviewing original data or suggesting update to its geometry, a compiler is asked to create a file of “IdE_Notes.txt” and leave short description regarding the updates done.

## Supplementary information

Supplementary Table 1

## Data Availability

SURE 2.0 database is available at Zenodo^3^.
